# The dosimetric impact of implants on the spinal cord dose during stereotactic body radiotherapy

**DOI:** 10.1186/s13014-016-0649-z

**Published:** 2016-05-25

**Authors:** Gozde Yazici, Sezin Yuce Sari, Fazli Yagiz Yedekci, Altug Yucekul, Sumerya Duru Birgi, Gokhan Demirkiran, Melis Gultekin, Pervin Hurmuz, Muharrem Yazici, Gokhan Ozyigit, Mustafa Cengiz

**Affiliations:** Department of Radiation Oncology, Hacettepe University, Faculty of Medicine, Ankara, Turkey; Department of Orthopedics and Traumatology, Hacettepe University, Faculty of Medicine, Ankara, Turkey

**Keywords:** Stereotactic body radiotherapy, Spinal implant

## Abstract

**Background:**

The effects of spinal implants on dose distribution have been studied for conformal treatment plans. However, the dosimetric impact of spinal implants in stereotactic body radiotherapy (SBRT) treatments has not been studied in spatial orientation. In this study we evaluated the effect of spinal implants placed in sawbone vertebra models implanted as in vivo instrumentations.

**Methods:**

Four different spinal implant reconstruction techniques were performed using the standard sawbone lumbar vertebrae model; 1. L2-L4 posterior instrumentation without anterior column reconstruction (PI); 2. L2-L4 anterior instrumentation, L3 corpectomy, and anterior column reconstruction with a titanium cage (AIAC); 3. L2-L4 posterior instrumentation, L3 corpectomy, and anterior column reconstruction with a titanium cage (PIAC); 4. L2-L4 anterior instrumentation, L3 corpectomy, and anterior column reconstruction with chest tubes filled with bone cement (AIABc). The target was defined as the spinous process and lamina of the lumbar (L) 3 vertebra. A thermoluminescent dosimeter (TLD, LiF:Mg,Ti) was located on the measurement point anterior to the spinal cord. The prescription dose was 8 Gy and the treatment was administered in a single fraction using a CyberKnife® (Accuray Inc., Sunnyvale, CA, USA). We performed two different treatment plans. In Plan A beam interaction with the rod was not limited. In plan B the rod was considered a structure of avoidance, and interaction between the rod and beam was prevented. TLD measurements were compared with the point dose calculated by the treatment planning system (TPS).

**Results and discussion:**

In plan A, the difference between TLD measurement and the dose calculated by the TPS was 1.7 %, 2.8 %, and 2.7 % for the sawbone with no implant, PI, and PIAC models, respectively. For the AIAC model the TLD dose was 13.8 % higher than the TPS dose; the difference was 18.6 % for the AIABc model. In plan B for the AIAC and AIABc models, TLD measurement was 2.5 % and 0.9 % higher than the dose calculated by the TPS, respectively.

**Conclusions:**

Spinal implants may be present in the treatment field in patients scheduled to undergo SBRT. For the types of implants studied herein anterior rod instrumentation resulted in an increase in the spinal cord dose, whereas use of a titanium cage had a minimal effect on dose distribution. While planning SBRT in patients with spinal reconstructions, avoidance of the rod and preventing interaction between the rod and beam might be the optimal solution for preventing unexpectedly high spinal cord doses.

## Introduction

Bone is the third most common site of metastatic cancer, and bone metastasis occurs in approximately 70 % of patients with metastatic breast cancer and prostate cancer [1]. The axial skeleton is the most frequently affected site [2–4]. Proper treatment of bone metastasis requires interdisciplinary care, and includes analgesics, corticosteroids (particularly in patients with spinal cord compression), hormonal therapy, bisphosphonates, surgery, chemotherapy, radiotherapy (RT), radiopharmaceuticals, or any combination of these.

Conventional external beam RT provides significant palliation of painful bone metastasis in 50–80 % of patients, but only 33 % of patients achieve complete pain relief at the treated site [5]. Studies have shown equivalent pain relief for various RT protocols, such as 30 Gy in 10 fractions, 24 Gy in 6 fractions, 20 Gy in 5 fractions, and a single 8-Gy fraction [6, 7]; however, the need for re-irradiation because of recurrent pain was higher in the single fraction arm, as compared to the fractionated protocols (20 % vs. 8 %). Hartsell et al. [7] evaluated patients with spinal metastasis separately and only 61 % of patients experienced partial or complete pain relief at 1 month post-treatment. Such studies highlight the need for improved outcomes in patients with spinal metastasis. Increasing the RT dose to the target is a legitimate option for increasing control rate; however, spinal cord tolerance is the limiting factor for increasing the delivered dose using conventional RT techniques.

Stereotactic body radiotherapy (SBRT) is an innovative treatment option for spinal metastasis. SBRT facilitates the delivery of a higher dose of radiation precisely to the target while limiting the dose to the spinal cord with rapid dose fall-off. Clinical studies on SBRT reported that it is an efficacious technique for pain control, with a rapid onset of response (as early as within 24 h) [8–10]. Additional experience with SBRT for spinal metastasis suggests a dose-response relationship, with an increase in pain relief—particularly at doses ≥16 Gy [10]. The major concern is the dose to the spinal cord, and the spinal dose constraint of 10 Gy to 10 % of the spinal cord defined in a maximum of 6 mm above and below the radiosurgery target is most commonly reported [11].

As SBRT is increasingly used in RT clinics for spinal metastases, patients with implants in the treatment field are often encountered. The spatial orientation of the implants in the vertebra is alike in patients with same implantation techniques. When a cage is used for vertebral column reconstruction the minimum distance between the cage and the spinal cord is 5 mm. The distance between the rods and the cord is more than 2 cm. The present study aimed to determine the dosimetric effect of spinal implants on the dose received by the spinal cord, in a sawbone model where the configuration of the implants could be simulated as in vivo.

## Materials and Methods

**Fig. 1:**
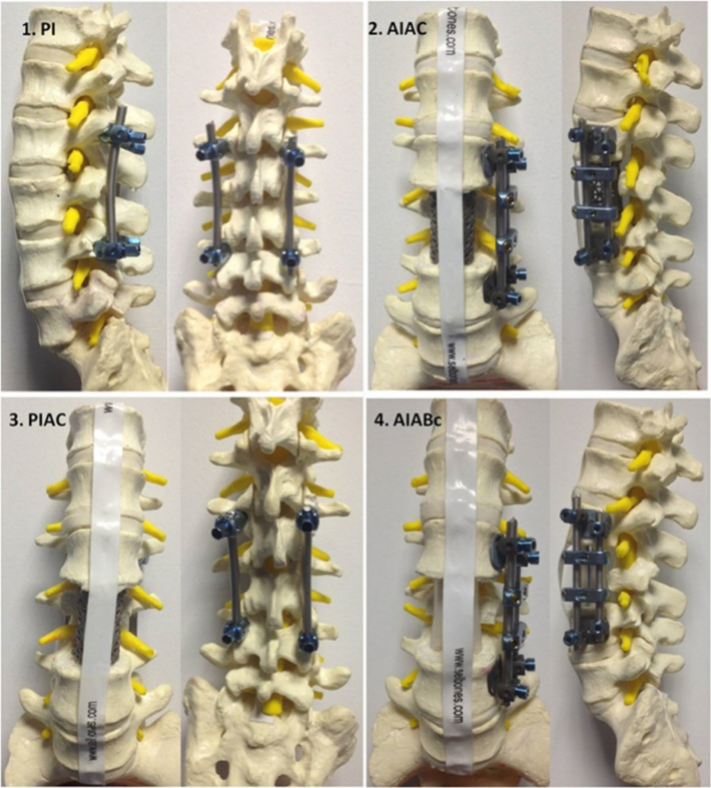
Spinal implant reconstruction models on the standard sawbones of lumbar vertebrae. 1. PI: Posterior instrumentation. 2. AIAC: Anterior instrumentation and anterior column reconstruction with use of a titanium cage. 3. PIAC: Posterior instrumentation and anterior column reconstruction with use of a titanium cage. 4. AIABc: Anterior instrumentation and anterior column reconstruction with use of chest tubes filled with bone cement

In this study 4 different spinal implant reconstruction models were employed on the standard sawbones of lumbar vertebrae, as follows: 1. Posterior instrumentation (PI); 2. Anterior instrumentation and anterior column reconstruction with use of a titanium cage (AIAC); 3. Posterior instrumentation and anterior column reconstruction with use of a titanium cage (PIAC); 4. Anterior instrumentation and anterior column reconstruction with use of chest tubes filled with bone cement (AIABc) (Fig. 1). We placed the vertebra models as if the patient was lying in supine position. We delineated the target volume as the spinous process and lamina of the lumbar (L) 3 vertebra in compliance with the definition in RTOG 0631 study [12]. We used the titanium CD Horizon Legacy 5.5 (Medtronic Sofamor Danek, Minneapolis, Minnesota USA) for posterior instrumentation, the CD Horizon Antares system (Medtronic Sofamor Danek, Minneapolis, Minnesota, USA) for anterior instrumentation, the Pyramesh (Medtronic Sofamor Danek, Minneapolis, Minnesota, USA) for vertebral body replacement, and polymethylmethacrylate cement (Surgical Simplex P, Howmedica, Limerick, Ireland).

**Fig. 2:**
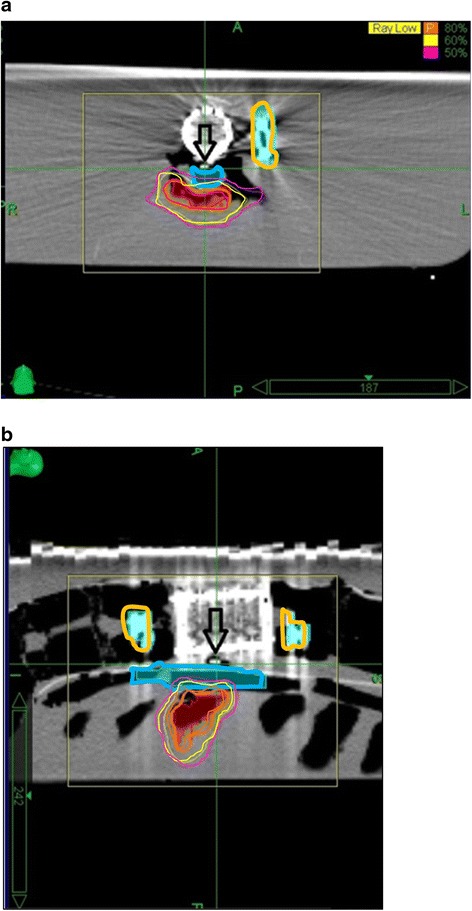
**a** and **b**. TLD (Thermoluminescent dosimeter) placement in the AIAC model. Figure 2A shows the axial and 2B shows the sagittal views in treatment position. The arrow shows the TLD behind the vertebral body, anterior to the spinal cord. The blue-outlined and orange-outlined structures represent the spinal cord and rods, respectively. The red structure represents the target volume. The orange, yellow and pink lines represent 80 %, 60 %, and 50 % isodoses respectively

To simulate the soft tissue around the vertebrae these models and a standard sawbone were placed in water separately. CT images (1.25-mm thick) of the sawbones placed in water were obtained using a GE High-Speed NX/I CT simulator (GE Medical System, Milwaukee WI, USA). Before performing each CT scan, a 4.5x0.89 mm thermoluminescent dosimeter (TLD, LiF:Mg,Ti) was placed in the space between the spinal cord and the posterior border of the vertebral body, just anterior to the structure representing the spinal cord so as only to define the point of measurement in the CT slices and to determine the exact dose at that point in the treatment planning system (TPS) (Fig. 2). This TLD was used only to guide us to measure the point dose at and around that point in the TPS. The target at the spinous process and lamina of the L3 vertebra was delineated according to the Radiation Therapy Oncology Group (RTOG) 0631 trial protocol when the metastasis involved only the posterior element [12]. The spinal cord was also delineated as the organ at risk (OAR). To obtain a homogenous dose distribution around the point of measurement we defined the TLD with a 0.5-cm margin in all directions as a structure. The planning CT (GE BrightSpeed 16 Slice CT, GE HealthCare, USA) does not have a software for metal artifact reduction, so we did not perform any compensation for metal artifacts.

### Treatment Planning

**Fig. 3:**
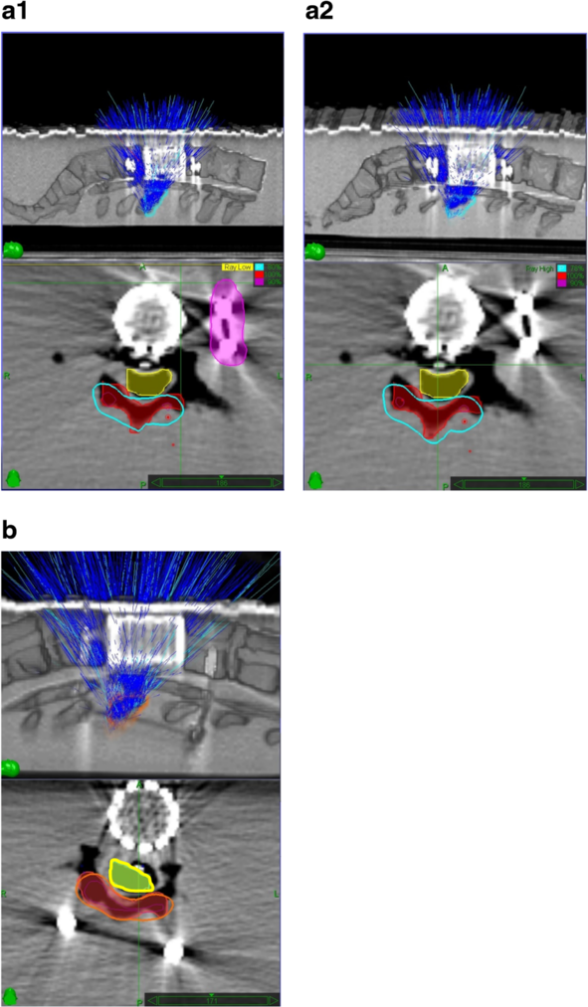
**a** (1,2) and **b**. The treatment plans of AIAC (3A1 and 3A2) and PIAC (3B); above: sagittal view, below: transverse view. Figure 3A1 and A2 shows the AIAC model with the interaction between the entering beams and rods blocked and unblocked, respectively. In Figure 3A1 and A2; yellow: spinal cord, red: target volume, cyan: 80 % isodose line, pink: blocked rod. In Fig. 3B; yellow: spinal cord, red: target volume, orange: 100 % isodose line

**Fig. 4:**
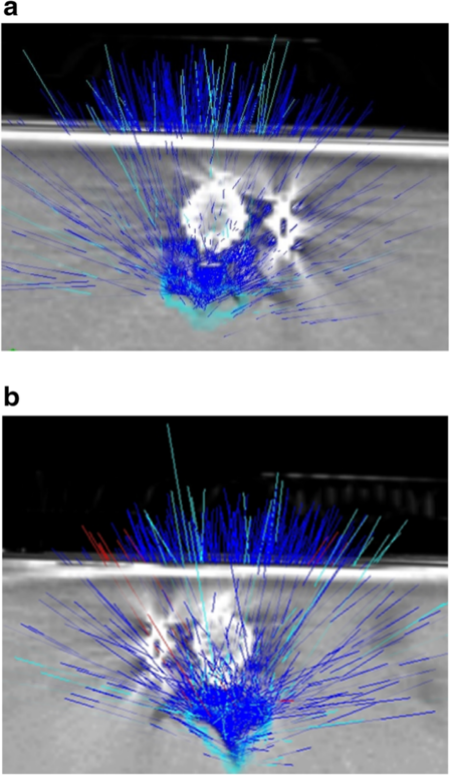
**a** and **b**. The axial view of the entering beams in AIAC models shown in Figure 3A1 and A2. Light blue rays are beam-on and dark blue rays are beam-off treatment positions, respectively. Figure 4A and B show the entering beams with the interaction between beams and rods blocked and unblocked, respectively

We used MultiPlan (Accuray Inc., Sunnyvale, CA, USA) inverse planning software for treatment planning and the prescription dose was 8 Gy. The treatments were administered in a single fraction using a CyberKnife® (Accuray Inc., Sunnyvale, CA, USA) (Figs. 3 and 4). For anterior instrumentation models 2 different plans were performed. In plan A beam interaction between the entering beams and the rod was not limited. In plan B the rod was considered a structure of avoidance and interaction between the rod and the entering beam was prevented. The TLDs used during the irradiation were different from the ones used during the CT scan. To minimize the potential error due to the localization of the TLD chips and dose gradients, we gave constraints to the TLD with a 0.5-cm margin around it. A region covered by 2 Gy with a 1.25 homogeneity index (HI) was obtained in 3 dimensions. Homogeneity index was defined as the maximum dose within target volume/prescribed dose, as proposed by RTOG [13]. As the TPS in the CyberKnife® does not allow to set a density override on metal artifact-affected regions, we did not perform any compensation for metal artifacts during treatment planning.

### TLD calibration

TLD 100 chips were calibrated with 2 Gy to a field of 10 cm × 10 cm with Varian Clinac DHX High Performance linear accelerator (Varian Medical Systems, Inc., Palo Alto, CA) using 6 MV photons, as this process is not practical with a CyberKnife® [14]. In total, 101 TLDs were placed in a specially designed water equivalent phantom (PMMA). Calibration factors were defined for each TLD.

### Dose measurement

The TLDs were encapsulated with paper and water proof tape in order to prevent them from the effects of water and other environmental factors. Each TLD location was labeled by a letter-number couple and irradiated. Pencil beam algorithm was used for dose calculation. Measurements were performed 3 times, and the mean was calculated. The annealing process started with annealing at 400 °C for 1 hour and 100 °C for 2 hours prior to irradiation, and preheating at 140 °C for 10 seconds and heating up to 280 °C at a rate of 5 °C s^-1^ after following irradiation, the same way as Bassinet et al. performed (12). The results were compared with the point dose calculated by the TPS. The following formula was used to calculate variations in dose by percentage:

$$ \left[\mathrm{D}\mathrm{o}\mathrm{s}{\mathrm{e}}_{\mathrm{TLD}}\hbox{--}\ \mathrm{D}\mathrm{o}\mathrm{s}{\mathrm{e}}_{\mathrm{TPS}}\right]\kern0.1em /\kern0.1em \mathrm{D}\mathrm{o}\mathrm{s}{\mathrm{e}}_{\mathrm{TPS}}, $$

where Dose_TLD_ is the dose measured at each TLD in each model and Dose_TPS_ is the dose calculated by the TPS.

## Results

Mean doses administered to the TLDs, the TPS-calculated dose, and differences between them are shown in Table 1. TLD measurements and the TPS dose differed by 1.7, 2.8, and 2.7 % for the sawbone model with no implant, the PI model, and the PIAC model, respectively. These findings show that the titanium cage had a minimal effect on dose distribution, as the percentages of difference corresponded to the clinical acceptability range of ±5 % [15]; therefore, beam interaction with the cage was not limited—that is to say the cage was not blocked. For the AIAC model the TLD dose was 13.8 % higher than the TPS dose. Furthermore, the difference was 18.6 % for the AIABc model. Taking these dose differences into consideration, the rods were blocked for plan B. In plan B for the AIAC and AIABc models the TLD doses were 2.5 and 0.9 % higher, respectively, than the TPS dose. Blocking the rods reduced the percentage of difference between TLD measurements and the TPS dose.

**Table 1:** TLD and TPS doses for each model

Models	TPS dose (cGy)	1st measurement (cGy)	2nd measurement (cGy)	3rd measurement (cGy)	Mean	Difference (%)
No implant	230	233	234	236	234	1.7
PI	245	235	263	258	252	2.8
PIAC	220	207	224	218	216	2.7
AIAC	230	254	264	270	262	13.9
AIAC- Plan B	235	235	225	228	229	2.5
AIABc	350	418	414	413	415	18.6
AIABc-Plan B	230	234	227	236	232	0.9

*TLD* Thermoluminescent dosimeter, *TPS*, Treatment planning system, *PI* posterior instrumentation, *AIAC* anterior instrumentation and anterior column reconstruction with use of a titanium cage, *PIAC* posterior instrumentation and anterior column reconstruction with use of a titanium cage, *AIABc* anterior instrumentation and anterior column reconstruction with use of chest tubes filled with bone cement

## Discussion

SBRT for the spine has been used at RT clinics in patients with spinal metastases with increasing frequency. The spinal cord dose constraint for SBRT is defined as 10 Gy in a single fraction, and because of the possible devastating results of a spinal cord overdose, this constraint is strictly followed. In the present study the effect of spinal implants on the spinal cord dose during SBRT for the treatment of spinal metastases was evaluated using morphologic replicas of vertebrae and implants that can be seen in patients in routine practice, and spinal cord doses ≤18.6 % higher than TPS doses were observed.

Patients with an impending fracture or a pathologic fracture, spinal instability, progressive spinal cord compression (SCC), progressive spinal deformity, or recurrent symptoms following RT require surgical intervention [16, 17]. Spinal cord compression is observed in approximately 20 % of patients with vertebral body metastases [18]. In cases of collapsed vertebral bodies vertebroplasty is the treatment of choice. Several types of surgical techniques can be used for vertebral body stabilization. Instrumentation with a rod can be performed posteriorly or anteriorly with a cage—with or without anterior column reconstruction. Patchell et al. [19] compared surgery to RT alone in patients with SCC, and reported that surgery was superior to RT for all endpoints, such as fecal continence, overall survival, and the need for pain killers. The combination of surgery and RT resulted in a greater likelihood of walking in patients who were paraplegic prior to surgery. Even partial resection of spinal metastasis increased the effectiveness of RT [19]. Ghogawala et al. [20] reported that wound complications occurred more frequently when surgery followed RT, as compared to when surgery was performed prior to RT (32 % vs. 12 %) ; therefore, surgery followed by RT is the recommended treatment for patients with SCC [19].

The most important problem with postoperative RT is radiation scatter due to implants. As implants are made of high atomic number materials, such as titanium, they cause dosimetric uncertainty due to beam attenuation and scatter [21]. The dosimetric effect of hip implants in pelvic RT has been studied. Bahreyni Toossi et al. [22] studied electron and neutron contamination of 4 different hip prosthesis varying in composition, including cobalt-chromium-molybdenum, stainless steel, titanium-alloy, and titanium. They observed an increase in electron and neutron contamination in all 4 implants. On the other hand, Schneider et al. measured neutron doses owing to titanium-alloy prostheses during photon and proton therapy and found no impact of prostheses on neutron contamination [23]. In another study based on 6 MV photons, dose inhomogeneity of 10 % was calculated in the vicinity of the implant, whereas in areas away from the implant dose homogeneity was within 5 % of the prescribed dose [24].

The American Association of Physicists in Medicine (AAPM) Task Group 63 published a report to inform the radiation oncology community of the problems associated with metal implants in the treatment field, and provide recommendations related to treatment planning and delivery in patients with hip prostheses [21]. They proposed beam arrangements that completely or partially avoided prostheses; however, what was proposed had the potential to increase the dose to the OARs.

There are also dosimetric studies performed with spinal implants. Wang et al. [25] performed a dosimetric study based on small fields ranging from 2x2 to 5x5 cm, and used 5-mm thick titanium rods embedded in water. They reported that the dose was 6 % higher at the intersection point of the rod and water due to backscattered electrons, and was 7 % lower in the shadow of the rod because of photon attenuation. Liebross et al. [26] studied the dose perturbation of screws and rods used for spinal implants. They used a 20x20-cm field and observed a dose reduction behind the rods. Son et al. [27] measured doses in the space between 2 titanium screws and reported the dose was 1.9 % lower, as measured via an ionization chamber.

The spatial relationship that parts of implants have with each other and with the OARs was not taken into consideration in the studies mentioned above. To overcome this limitation Pekmezci et al. performed 4 spinal implant reconstructions in sawbone spine models to evaluate the effects of implants on dose distribution for a single field (postero-anterior) technique using a cobalt-60 unit and a 6-MV linear accelerator [28]. Posterior instrumentation resulted in a 5 % lower dose, whereas the anterior instrumentation models did not have a significant effect on dose distribution.

The CyberKnife® is an image-guided stereotactic radiosurgery and SBRT device that facilitates tumor treatment by generating a large number of direction points (typically 1000-6000); however, use of a high number of beams at different angles causes complex interactions between each beam and the implants. We hypothesized that these complex interactions between beams and implants may cause an unpredictable and totally different dosimetric effect than the interactions between beams and implants that occur with conventional treatment plans. It has been shown that the TPS generally calculates wrong dose values in the vicinity of metal artifacts. This led to a standard procedure of setting the density to a value that would correspond to the soft tissue obscured by the artifacts during treatment planning [29]. However, as both our planning CT and the TPS of CyberKnife® do not allow this option, we could not perform any compensation for the metal artifacts.

During stereotactic RT treatments in CyberKnife® patients are usually treated in supine position, and this device allows beam delivery from the anterior and lateral directions. Therefore, the anterior rod placement in this position results in an interaction between the rod and entering beams. However, in posterior instrumentations the rods are located in the path of exit beams. The present findings show that the interaction with the entering beams caused unacceptably high spinal doses. Furthermore, use of a titanium cage or chest tubes filled with cement placed anterior to the spinal cord did not have a significant dosimetric effect. This observation provides a guide in clinical practice because describing all implants as structure of avoidance according to the AAPM task group’s recommendation limits the angles that can be used with SBRT profoundly. Limiting the number of angles can result in unacceptably high doses in the adjacent organs. Based on the present findings, we suggest that only rods—when placed anterior to the spinal cord—should be considered structures of avoidance when using a TPS to calculate the absorbed dose.

## Conclusions

Spinal implants may be present in the treatment field in patients scheduled to undergo SBRT. For the types of implants studied herein anterior rod instrumentation resulted in an increase in the spinal cord dose, whereas use of a titanium cage had a minimal effect on dose distribution. While planning SBRT in patients with spinal reconstructions, avoidance of the rod and preventing interaction between rod and entering beam might be the optimal solution for preventing unexpectedly high spinal cord doses.
